# A Multiobjective Interval Programming Model for Wind-Hydrothermal Power System Dispatching Using 2-Step Optimization Algorithm

**DOI:** 10.1155/2014/825216

**Published:** 2014-05-07

**Authors:** Kun Ren, Qu Jihong

**Affiliations:** ^1^Institute of Water Resources and Hydro-electric Engineering, Xi'an University of Technology, Xi'an 710048, China; ^2^North China University of Water Resources and Electric Power, Zhengzhou 450011, China

## Abstract

Wind-hydrothermal power system dispatching has received intensive attention in recent years because it can help develop various reasonable plans to schedule the power generation efficiency. But future data such as wind power output and power load would not be accurately predicted and the nonlinear nature involved in the complex multiobjective scheduling model; therefore, to achieve accurate solution to such complex problem is a very difficult task. This paper presents an interval programming model with 2-step optimization algorithm to solve multiobjective dispatching. Initially, we represented the future data into interval numbers and simplified the object function to a linear programming problem to search the feasible and preliminary solutions to construct the Pareto set. Then the simulated annealing method was used to search the optimal solution of initial model. Thorough experimental results suggest that the proposed method performed reasonably well in terms of both operating efficiency and precision.

## 1. Introduction

Due to the increasingly serious energy and environmental problems, renewable energy has become an important research topic, and extensive work has been conducted to advance the technologies of power generation systems based on various renewable sources, such as solar energy, geothermal, biomass, fuel cell, and industrial waste heat [1–4]. Wherein, wind power technology is one of the fastest growing and the most mature technologies in renewable energy. In the wind-hydrothermal power system, the purpose of the dispatching is to make a reasonable decision for power plant output based on the optimal scheduling goal and on the premise of system energy balance and operation constraint. In the research of power system optimization scheduling problem, two key problems should be solved: a proper optimization model and accurate and efficient solution to the model.

Firstly, in the aspect of establishing model, as the prediction for hydroplant runoff and wind power output and power load is inaccurate, deterministic model [5, 6] to simulate those uncertain data is irrational. So many scholars used stochastic programming [7–9] and fuzzy programming [10–13] to draw up those uncertainty data. However, the membership functions in fuzzy programming model and precise probability distribution of random variables in the stochastic programming model are usually difficult to obtain accurately. Therefore, the above two kinds of modeling method are limited in practical applications.

For the past few years, another modeling method using interval programming [14] to describe the uncertainty number have been received attention. Interval programming makes the uncertainty number into interval number and only needs to determine their upper and lower boundaries. In practical applications, to derive the upper and lower boundaries of the uncertainty number is much easier than the probability distribution of random variables in the stochastic programming and the membership function in fuzzy programming. Random variable in the stochastic programming and fuzzy number in the fuzzy programming can be transformed into intervals through confidence level and assembly level, respectively [15, 16]. Therefore, the intervals become other very important and practical forms of uncertainty information and have been widely used in many fields such as economic profit maximization [17], design of wind [18], and design of automobile [19]. Interval model is also used to model the power system dispatching but only for the single-objective optimization [20].

Secondly, in the other aspect, methods for solving the model have always been the hot spot in the current study. According to the type of the objective function, solving the optimization problem can be divided into two categories: linear and nonlinear. A series of methods represented by linear programming [21] can quickly and accurately solve the optimization problem, but they require very strict form of the objective function. So, these methods cannot directly solve the multiobjective model for power generation systems. Another kind of nonlinear algorithm such as gradient descent and Powell speedup search has obtained the good effect on the small-scale optimization problem. For large-scale nonlinear optimization problem, heuristic search algorithm has received more attention in recent years. Heuristic search algorithms such as various modified forms of genetic algorithms [22], evolutionary programming [23, 24], particle swarm optimization [13], and simulated annealing [25] have the capability of global search. Although the searching does not get the accurate optimal solutions quickly, their search strategy can quickly draw near the optimal solution. Thus, this algorithm has been extensively used for many applications.

This paper proposed a method using the interval to describe the uncertain number in wind-hydrothermal power system. A multiobjective optimization scheduling decision model that contains interval number is established to assist real-time scheduling. In order to reduce the complexity of multiobjective model with interval parameters, a 2-step optimal method is established by utilizing the linear programming and simulated annealing.

The structure of the paper is organized as follows. In Section 2, uncertain information range simulation is introduced. In Section 3, a multiobjective scheduling model is developed based on interval simulation. In Section 4, solving method of interval multiobjective model is proposed. The experimental results are analyzed and discussed in Section 5. Some conclusions and further studies are discussed and generalized in Section 6.

## 2. Interval Simulation of Uncertainty Information

If the upper and lower bounds of an uncertainty variable *U* are known, we can use an interval number as shown in (1) to describe the uncertainty variables:

(1) $$\begin{array}{c} U⟹\left[U_{u},U_{l}\right], \end{array}$$

where  *U*_*u*_ and *U*_*l*_ are upper and lower limits of *U*, respectively. If *U*_*u*_ is equal to *U*_*l*_, *U* becomes a certainty number as shown in

(2) $$\begin{array}{c} U=U_{u}=U_{l}. \end{array}$$

In the wind-hydrothermal power system, it is necessary to predict hydroplant runoff, wind power output, and power load. Influenced by prediction accuracy, deterministic predicting results often deviate from the actual value. Therefore, deterministic model would fail to meet the needs of the reasonable modeling. The result described by interval number reflects the uncertainty of results more objectively than the deterministic number and this method has usually been applied in the prediction field [26].

In the scheduling period, hydroplant runoff, wind power output, and power load can be simulated by interval number as follows:

(3) $$\begin{array}{c} P_{lf}^{t}⟹\left[P_{lfu}^{t},P_{lfl}^{t}\right],\;\;P_{Wf}^{t}⟹\left[P_{Wfu}^{t},P_{Wfl}^{t}\right],\\ q_{f}^{t}⟹\left[q_{fu}^{t},q_{fl}^{t}\right], \end{array}$$

where  *P*_*lf*_^*t*^ is the forecast value of power load at time *t*, *P*_*wf*_^*t*^ is the forecast value of wind power output at time *t*, and *q*_*f*_^*t*^ is the forecast value of hydroplant runoff at time *t*.

Deterministic model will generally produce the same solutions from a given initial condition. In actual operation, in order to deal with the deviation of wind power prediction output and the load prediction results, power system dispatching departments must adjust deterministic solving result from deterministic model in real time so as to ensure safe and stable operation of the power system. So it is unreasonable to model the output of a power plant in the system by deterministic number. In this paper, we simulate the output of a power plant by interval number as it is shown in the following function:

(4) $$\begin{array}{c} P_{H}^{t}⟹\left[P_{Hu}^{t},P_{Hl}^{t}\right],\;\;P_{T}^{t}⟹\left[P_{Tu}^{t},P_{Tl}^{t}\right],\\ P_{W}^{t}⟹\left[P_{Wu}^{t},P_{Wl}^{t}\right], \end{array}$$

where  *P*_*H*_^*t*^ is the output value of hydroplant at time *t*, *P*_*T*_^*t*^ is the output value of thermal plant at time *t*, and *P*_*w*_^*t*^ is the output value of wind plant at time *t*.

## 3. Multiobjective Model Based on Interval Simulation 

### 3.1. Multiobjective Function Based on Interval Simulation

The wind-hydrothermal power system is simulated by a deterministic multiobjective model. *f*_1_ is the system cost to purchase power, *f*_2_ is the coal consumption of thermal plant, *f*_3_ is the water deprivation of hydroplant, *f*_4_ is the emission of pollution gas of thermal plant, and *f*_5_ is the abandon value of wind plant:

(5) $$\begin{array}{c} \min\{\begin{array}{cc} f_{1}=\overset{T}{\underset{t=1}{\sum }}(\overset{N_{H}}{\underset{j=1}{\sum }}C_{H}^{t}P_{H,j}^{t}+\overset{N_{T}}{\underset{i=1}{\sum }}C_{T}^{t}P_{T,i}^{t} & \\ \;\;\;\;\;+\;\overset{N_{K}}{\underset{k=1}{\sum }}C_{W}^{t}P_{W,k}^{t}), & \\ f_{2}=\overset{T}{\underset{t=1}{\sum }}\overset{N_{T}}{\underset{\;i=1}{\sum }}\left[a_{i}{\left(P_{T,i}^{t}\right)}^{2}+b_{i}P_{T,i}^{t}+c_{i}\right], & \\ f_{3}=\overset{T}{\underset{t=1}{\sum }}\overset{N_{H}}{\underset{\;j=1}{\sum }}Q_{j}^{t}=\overset{T}{\underset{t=1}{\sum }}\overset{N_{H}}{\underset{\;j=1}{\sum }}\left(\frac{P_{H,j}^{t}}{0.00981\eta_{j}H_{j}^{t}}\right), & \\ f_{4}=\overset{T}{\underset{t=1}{\sum }}\overset{N_{T}}{\underset{\;i=1}{\sum }}\left[\mu_{i}{\left(P_{T,i}^{t}\right)}^{2}+\upsilon_{i}P_{T,i}^{t}+\omega_{i}\right], & \\ f_{5}=\overset{T}{\underset{t=1}{\sum }}\overset{N_{K}}{\underset{\;k=1}{\sum }}\left(P_{W,k}^{t}-P_{Wa,k}^{t}\right). & \end{array} \end{array}$$

Through interval simulation of uncertain value, function (5) can be changed into function (6):

(6) $$\begin{array}{c} \min\{\begin{array}{cc} f_{1}=\overset{T}{\underset{t=1}{\sum }}(\overset{N_{H}}{\underset{j=1}{\sum }}C_{H}^{t}\left[P_{H\text{up},j}^{t},P_{H\text{low},j}^{t}\right] & \\ \;\;\;\;\;+\;\overset{N_{T}}{\underset{i=1}{\sum }}C_{T}^{t}\left[P_{T\text{up},i}^{t},P_{T\text{low},i}^{t}\right] & \\ \;\;\;\;\;+\;\overset{N_{K}}{\underset{k=1}{\sum }}C_{W}^{t}\left[P_{W\text{up},k}^{t},P_{W\text{low},k}^{t}\right]), & \\ f_{2}=\overset{T}{\underset{t=1}{\sum }}\overset{N_{T}}{\underset{\;i=1}{\sum }}(a_{i}{\left[P_{T\text{up},i}^{t},P_{T\text{low},i}^{t}\right]}^{2} & \\ \;\;\;\;\;\;\;+\;b_{i}\left[P_{T\text{up},i}^{t},P_{T\text{low},i}^{t}\right]+c_{i}), & \\ f_{3}=\overset{T}{\underset{t=1}{\sum }}\overset{N_{H}}{\underset{\;j=1}{\sum }}Q_{j}^{t} & \\ \;=\overset{T}{\underset{t=1}{\sum }}\overset{N_{H}}{\underset{\;j=1}{\sum }}\left(\frac{\left[P_{H\text{up},j}^{t},P_{H\text{low},j}^{t}\right]}{0.00981\eta_{j}H_{j}^{t}}\right), & \\ f_{4}=\overset{T}{\underset{t=1}{\sum }}\overset{N_{T}}{\underset{\;i=1}{\sum }}(\mu_{i}{\left[P_{T\text{up},i}^{t},P_{T\text{low},i}^{t}\right]}^{2} & \\ +\upsilon_{i}\left[P_{T\text{up},i}^{t},P_{T\text{low},i}^{t}\right]+\omega_{i}), & \\ f_{5}=\overset{T}{\underset{t=1}{\sum }}\overset{N_{K}}{\underset{\;k=1}{\sum }}(\left[P_{Wf\text{up},k}^{t},P_{Wf\text{low},k}^{t}\right] & \\ -\left[P_{Wa\text{up},k}^{t},P_{Wa\text{low},k}^{t}\right]), & \end{array} \end{array}$$

where  *N*_*W*_, *N*_*H*_, and *N*_*T*_ are the numbers of wind plant, hydroplant, and thermal plant, respectively. *C*_*H*_^*t*^ is the price of hydroelectricity at time *t*. *C*_*T*_^*t*^ is the price of thermal electricity at time *t*. *C*_*W*_^*t*^ is the price of wind electricity at time *t*. *a*_*i*_(*P*_*T*,*i*_^*t*^)^2^ + *b*_*i*_ *P*_*T*,*i*_^*t*^ + *c*_*i*_ represents the relation of quadratic function between coal consumption and thermal-plant output. *a*_*i*_, *b*_*i*_, *c*_*i*_ are coefficients of quadratic function. *μ*_*i*_, *υ*_*i*_, *ω*_*i*_ are coefficients of quadratic function between emissions of pollution gas of thermal plant and output of thermal plant. *P*_*T*,*i*_^*t*^ is the output of thermal plant at time *t*. *P*_*H*,*j*_^*t*^ is the output of hydroplant at time *t*. *P*_*W*,*k*_^*t*^ is the output of wind plant at time *t*. *Q*_*j*_^*t*^ is the water flow used to generate electric at time *t*. *η*_*j*_ is generation efficiency of hydroplant. *H*_*j*_^*t*^ is the net waterhead of hydroplant.

### 3.2. Constraint Condition of Electrical Power System

*(i) Constraint of Load Balancing*. Excluding the depletion of electric network, the load balance function is shown as in

(7) $$\begin{array}{c} \overset{N_{T}}{\underset{i=1}{\sum }}P_{T,i}^{t}+\overset{N_{H}}{\underset{j=1}{\sum }}P_{H,j}^{t}+\overset{N_{K}}{\underset{k=1}{\sum }}P_{W,k}^{t}=P_{l}^{t}. \end{array}$$

Through interval simulation of uncertain value, (7) can be derived into (8):

(8) ∑i=1NT[PTu,it,PTl,it]+∑j=1NH[PHu,jt,PHl,jt]+∑k=1NK[PWu,kt,PWl,kt]  =[Plut,Pllt].

*(ii) Constraint of Reservoir Capacity of Hydroplant.* Consider

(9) $$\begin{array}{c} {\underline{V}}_{j}\leq V_{j}^{t}\leq {\bar{V}}_{j},\;\left(j=1,2,\ldots ,N_{H};\;\;t\in T\right), \end{array}$$

where  ${\underline{V}}_{j}$ is the lower limit value of reservoir capacity. ${\bar{V}}_{j}$ is the upper limit value of reservoir capacity. *V*_*j*_^*t*^ is the reservoir capacity of hydroplant at time *t*.

*(iii) Constraint of Water Flow Which Is Used to Generate Electricity.* Consider

(10) $$\begin{array}{c} {\underline{Q}}_{j}\leq Q_{j}^{t}\leq {\bar{Q}}_{j}, \end{array}$$

where  ${\underline{Q}}_{j}$ is the minimum flow of hydroelectric generation set. ${\bar{Q}}_{j}$ is the maximum flow of hydroelectric generation set. *Q*_*j*_^*t*^ is the water flow which is used to generate electricity at time *t*. 

*(iv) Recurrence Relation of Water Amount of Hydroplant.* Consider

(11) $$\begin{array}{c} V_{j}^{t+1}=V_{j}^{t}+q_{j}^{t}-Q_{j}^{t}⟹V_{j}^{t+1}=V_{j}^{t}+\left[q_{u,j}^{t},q_{l,j}^{t}\right]-Q_{j}^{t}, \end{array}$$

where  *q*_*j*_^*t*^ is the value of hydroplant runoff at time *t*. 

*(v) Constraints of Output of Hydroplant and Thermal Plant.* Consider

(12) $$\begin{array}{c} {\underline{P}}_{H,j}\leq P_{H,j}^{t}\leq {\bar{P}}_{H,j},\;\;{\underline{P}}_{T,i}\leq P_{T,i}^{t}\leq {\bar{P}}_{T,i}. \end{array}$$

*(vi) Constraint of Climbing Ability of Thermal Power Generating Unit.* Consider

(13) |PT,it−PT,it+1|≤PTPP,i⟹|[PTu,it,PTl,it]−[PTu,it+1,PTl,it+1]|  ≤PTPP,i.

*P*_TPP,*i*_ is the maximum power which thermal plant can change from time *t* to *t* + 1.

*(vii) Water and Electricity Transformation Relationship.* Consider

(14) $$\begin{array}{c} P_{H,j}^{t}=0.00981\eta_{j}Q_{j}^{t}H_{j}^{t}. \end{array}$$

*H*_*j*_^*t*^ is the net waterhead of hydroplant:

(15) $$\begin{array}{c} H_{j}^{t}=h_{j}^{1}\left(V_{j}^{t-1}+q_{j}^{t-1}\right)-h_{j}^{2}\left(Q_{j}^{t-1}\right). \end{array}$$

*h*_*j*_^1^(·) is the relationship between upper pool elevation and capacity of reservoir. *h*_*j*_^2^(·) is the relationship between lower pool elevation and water flow:

(16) $$\begin{array}{c} q_{j}^{t-1}⟹\left[q_{u,j}^{t-1},q_{l,j}^{t-1}\right]. \end{array}$$

*(viii) Constraint of Spinning Reserve.* The interval simulation shows that the output of power plant contains spinning reserve. So the constraint of spinning reserve is not taken into account in this model.

*(ix) Constraint of Power Balance of Nodes.* Consider

(17) $$\begin{array}{c} P_{G,i}-P_{D,i}-\overset{n}{\underset{j=1}{\sum }}U_{i}Y_{ij}U_{j}\cos\delta_{ij}=0,\\ Q_{G,i}-Q_{D,i}-\overset{n}{\underset{j=1}{\sum }}U_{i}Y_{ij}U_{j}\sin\delta_{ij}=0. \end{array}$$

*n* is the number of nodes. *U*_*i*_ is voltage amplitude of the *i*th node. *δ*_*i*_ is the phase angle of the *i*th nodes. *Y*_*ij*_ and *α*_*ij*_ are amplitude and phase angle of transfer admittance from the *i*th node to the *j*th node:

(18) $$\begin{array}{c} \delta_{ij}=\delta_{i}-\delta_{j}-\alpha_{ij}. \end{array}$$

*P*_*G*,*i*_ is the active power of electric generator at the *i*th node. *P*_*D*,*i*_ is active power of load at the *i*th node. *Q*_*G*,*i*_ is wattless power of electric generator at *i*th node. *Q*_*D*,*i*_ is wattless power of load at the *i*th node.

*(x) Constraint of Voltage of Nodes.* Consider

(19) $$\begin{array}{c} {\underline{V}}_{i}\leq V_{i}\leq {\bar{V}}_{i},\;\left(i=1,2,\ldots ,n\right). \end{array}$$

*(xi) Constraint of Active Power and Wattless Power at Nodes.* Consider

(20) $$\begin{array}{c} {\underline{P}}_{G,i}\leq P_{i}\leq {\bar{P}}_{G,i},\\ {\underline{Q}}_{G,i}\leq Q_{i}\leq {\bar{Q}}_{G,i}. \end{array}$$

*(xii) Constraint of Phase Difference between Nodes.* Consider

(21) $$\begin{array}{c} \left|\delta_{i}-\delta_{j}\right|<{\left|\delta_{i}-\delta_{j}\right|}_{\max}. \end{array}$$

Superiority of interval model is as follows: output of the unit plan and standby output are established in one model. They meet the power system constraints, so it can avoid invalid standby that is checked by the power system constraints. Objective function values calculated through interval algorithm are interval numbers. Interval number reflects the uncertainty of empirical function values that are due to uncertainty of power plant output. An interval number quantizes uncertainty of quantitative function values. Thus, the foundation for the scheduling is established.

## 4. Solving Method of Interval Multiobjective Model

The existing heuristic search algorithm that solves multiobjective model is based on the Pareto-dominant relationship to compare the performance of different solutions. The gist of Pareto dominant is the objective function value of solutions. Compared to the deterministic value of traditional model, the objective function value, which is calculated by the model of this paper, is an interval number. So we use interval number dominant relationship to compare the performance of different solutions.

In order to further develop an efficient and precise method for solving the Pareto solution set, we use improved simulated annealing that adds the idea of NSGA-II [27] and linear program to solve the model. The interval algorithms, interval dominant relationship, and interval crowded degree are defined in the following sections.

### 4.1. Interval Algorithm

For the two interval numbers [*a*, *b*] and [*c*, *d*], *a* and *c* are lower bounds of interval number. *b* and *d* are upper bounds of interval number. *e* is real number:

(22) [a,b]+[c,d]=[a+c,b+d],[a,b]−[c,d]=[a−d,b−c],[a,b]×[c,d]=[a×c,b×d],[a,b]+e=[a+e,b+e],  [a,b]−e=[a−e,b−e],[a,b]×e=[a×e,b×e],a>e, b>e→yields[a,b]>e.

### 4.2. Interval Dominant Relationship

At present, there are many ranking methods of interval numbers [28]. We use interval dominant credibility to rank interval numbers [14].

**Definition 1**

For the two interval numbers [*a*, *b*] and [*c*, *d*], *a* and *c* are lower bounds of interval number. *b* and *d* are upper bounds of interval number. *b* − *a* and *d* − *c* are width of interval number. *γ* is interval dominant credibility; we defined *γ* as follows:

(23) γ=p([a,b]≥[c,d])=max⁡{1−max⁡(d−ab−a+d−c,0),0}.

### 4.3. Interval Crowded Degree

Crowding distance among the interval numbers with the same order value needs to be compared. So these solutions can have a good distribution, diversity, and ductility [29].

**Definition 2**

For the two interval numbers [*a*, *b*] and [*c*, *d*], *a* and *c* are lower bounds of interval number. *b* and *d* are upper bounds of interval number. The distance of the interval number is defined as follows:

(24) $$\begin{array}{c} d\left(\left[a,b\right],\left[c,d\right]\right)=\max\left\{\left|a-c\right|,\left|b-d\right|\right\}. \end{array}$$

### 4.4. The 2-Step Optimization

Due to the complexity of the multiobjective problem, the method using directly simulated annealing algorithm to solve the problem will lead to the quite different results because of different initial solutions. In addition, the variables exist in a complex, high-dimensional, and nonlinear space, and this causes serious difficulty to solve the problem precisely.

In order to solve the problem, a 2-step method is proposed in this paper. Firstly, the nonlinear objective function is simplified into a rectilinear objective function, as shown in

(25) min⁡f1=∑t=1T(∑j=1NHCHt[PHup,jt,PHlow,jt]+∑i=1NTCTt[PTup,it,PTlow,it]+∑k=1NKCWt[PWup,kt,PWlow,kt]).

Constraint condition include load balancing, power plant output, and so forth.

After getting the feasible result of linear programming, we order that Pareto solution set is equal to this approximate solution. Then we use simulated annealing algorithm to solve the problem precisely. Process of simulated annealing algorithm to solve the problem in this paper is listed in details as follows.

 Step 1: to initialize the parameters of simulated annealing algorithm, initial temperature *t*, temperature attenuation coefficient *dt*, and so forth.
 Step 2: generate the Pareto solution sets by repeatedly solving linear programming problems and calculate the objective function value of solution.
 Step 3: construct roulette with 0-1 range of Pareto set.
 Step 4: bet a solution from the roulette according to the probability and randomly disturb it.
 Step 5: replace and rank if the new solution is better than the original one or satisfies the Metropolis criterion. Otherwise, the solution is abandoned.
 Step 6: update the parameter *t* = *dt* · *t*.
 Step 7: terminate the algorithm if no further better results are achieved after certain iterations. Finally, output optimal solution and sort the Pareto solution set.

**Figure 1:**
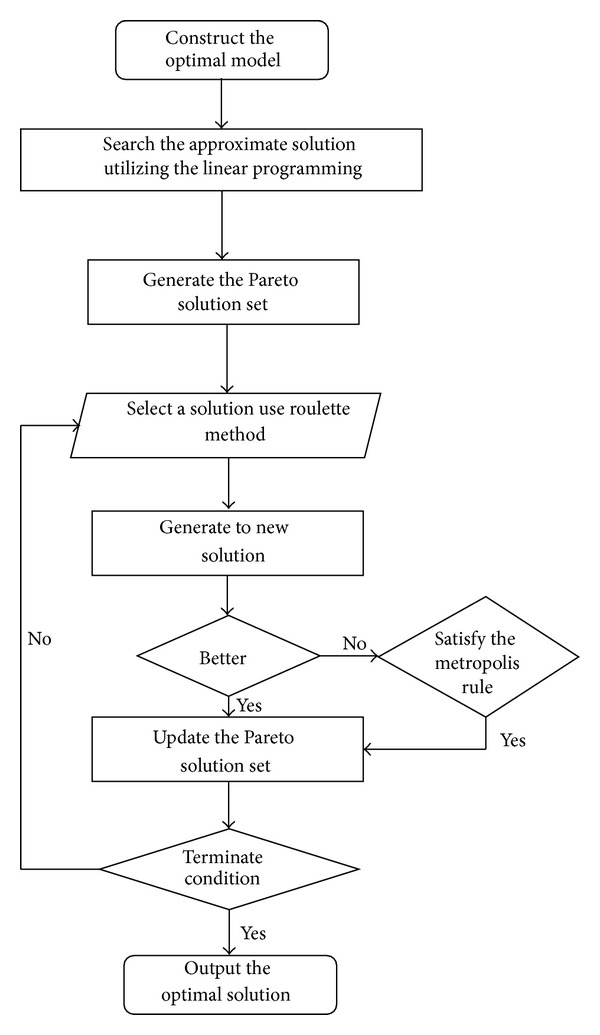
Flowchart of our algorithm.

In order to further clearly express the 2-step optimization algorithm, we illustrated the flowchart as shown in Figure 1.

## 5. Experiment 

We choose a power system with five thermal power plants, five hydropower stations, and a large wind farm in a province of China as an example to perform our experiment. Scheduling period is 24 hours, divided into 24 sessions, all the basic parameters of the thermal power plants are shown in Table 1 and the basic parameters of hydropower are shown in Table 2.

**Figure 2:**
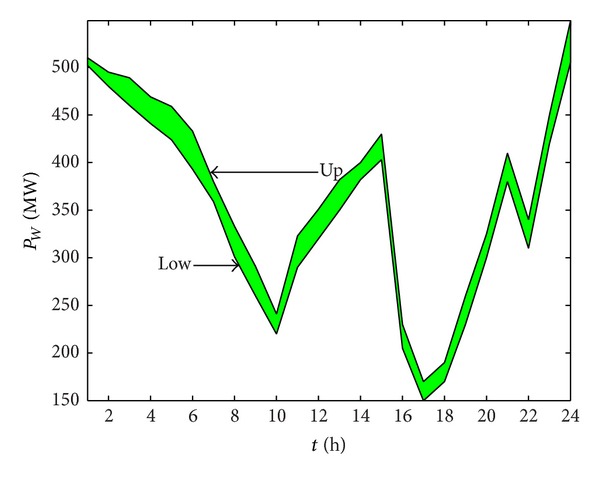
Prediction of the wind power output range.

**Figure 3:**
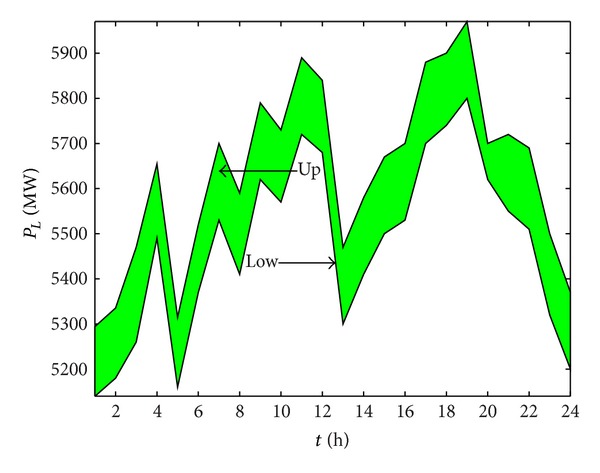
Prediction of the load interval value.

The predicted output ranges of the wind power plant at each time are shown in Figure 2; Figure 3 shows the prediction of load interval value.

**Figure 4:**
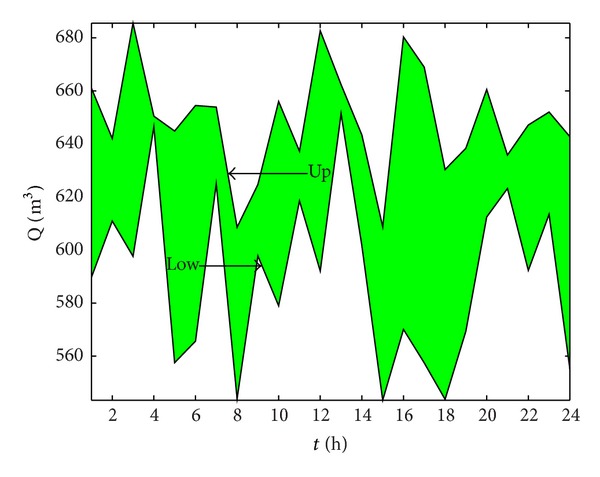
Prediction of hydropower plant runoff interval value.

In order to briefly show the effectiveness of our method, the runoffs of five hydropower plants are set at the same predicted internal value, as shown in Figure 4.

In the experiment, all of wind power output is received by the system. Hydroelectricity price is 0.25 RMB kW/h. Thermal power electricity price is 0.33 RMB kw/h. Wind power electricity price is 0.45 RMB kw/h. Interval dominant credibility is greater than 0.5. Initial parameters of simulated annealing are set as follows:

*t* = 2, *dt* = 0.98, *s* = 2, *k* = 100. (These parameters are got through running ten times our experiment.)

**Figure 5:**
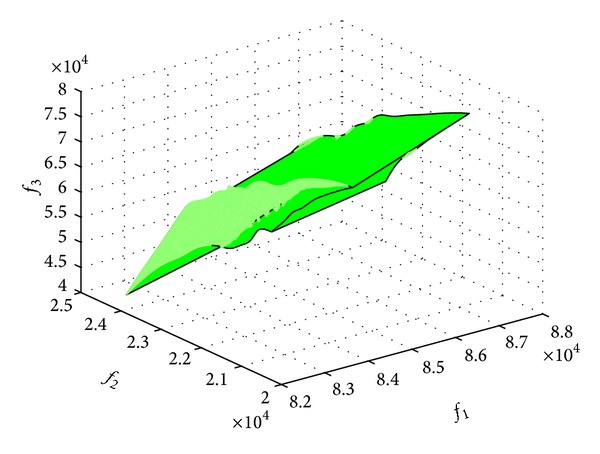
Pareto cube with three objects *f*_1_ *f*_2_ and *f*_3_.

Consider *f*_1_, *f*_2_, and *f*_3_; the Pareto cube of the three targets is shown in Figure 5.

**Figure 6:**
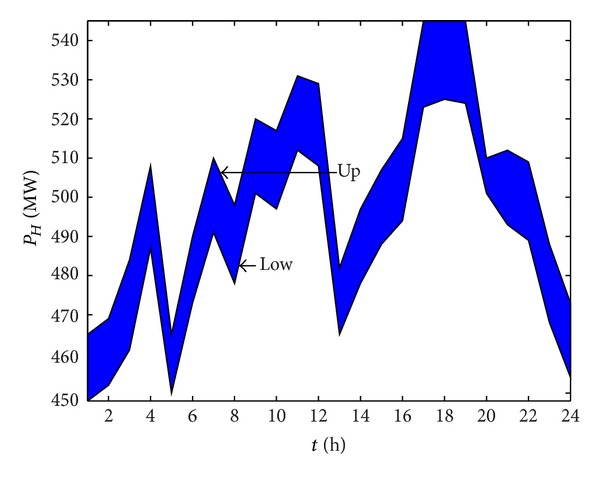
The optimal output range of Hydropower 1.

**Figure 7:**
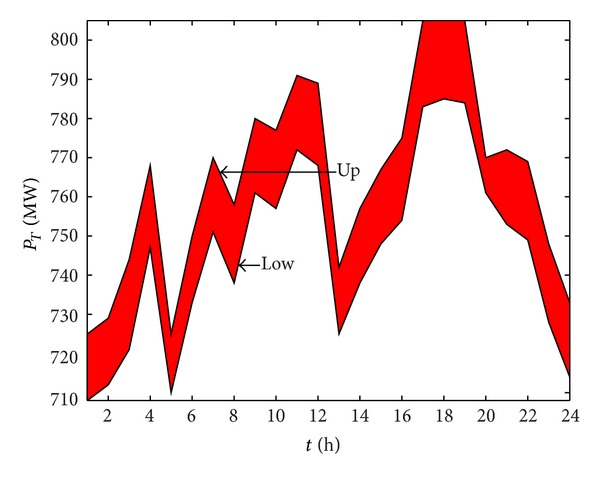
The optimal output range of thermal power plant 1.

Considering *f*_1_, *f*_2_, *f*_3_, and *f*_4_, we get the optimal Pareto set. By selecting one of the Pareto sets, the optimal solution of hydropower plant 1 is shown in Figure 6 and the output power is shown in Figure 7.  Table 3 shows the optimal output range of all power plants. Obviously, the trends of the two figures should be the same, and these also denote the accuracy of our model.

The average computational time for the problem is around 50 ms per iteration for the interval programming. In fact, we hardly get the results that satisfy the constraints if we simply use the simulated annealing algorithm without any strategy. In particular, the time complexity of nonlinear programming is presented with the increase in exponential growth trend.

After the optimal output range of each power plant in the every scheduling time was obtained, real-time scheduling only needed to consider the constraint of output range, load balancing, and the scheduling goal because the output range was satisfied with the all constraint of power system. Thus, the real-time scheduling model is simplified and convenient for real-time solution. The solution of real-time scheduling can satisfy the optimal decision in scheduling period because the output range of each power plant is the optimal decision in interval model.

## 6. Conclusion

In this paper, we presented an interval program model for wind power scheduling system. The model utilizing the interval theory is able to reasonably simulate the problem of wind-hydrothermal power system dispatching. The 2-step optimization method can solve the complex models efficiently. Experimental results showed that our method has a high precision and speed. Therefore, it is suitable to solve large-scale interval programming model. Further work will focus on the following: (i) combined model including probability and interval should be established and (ii) the solving method should be further improved. How to search the new solutions based on the Pareto set to enhance the performance of our approach will be a further study.

## Figures and Tables

**Table 1:** Basic parameters of thermal power plants.

Index	Capacity (MW)	Min output (MW)	*a*	*b*	*c*	*u*	*v*	*w*	*V* (MW/h)
1	1200	500	2.8 × 10^−6^	0.274	13.7	4.692	−6.374	5.065	48
2	1200	500	1.39 × 10^−5^	0.259	14.5	5.472	−6.812	5.723	60
3	600	280	6.11 × 10^−5^	0.279	6.35	4.312	−5.764	5.365	36
4	1200	480	8.3 × 10^−4^	0.269	14.1	5.836	−6.735	4.926	50
5	720	330	3.33 × 10^−5^	0.312	4.64	6.548	−6.264	5.569	40

**Table 2:** Basic parameters of hydropower stations.

Index	Capacity (MW)	Min Output (MW)	Max storage C (1 × 10^8^ m^3^)	Min storage C (1 × 10^8^ m^3^)	Initial storage C (1 × 10^8^ m^3^)	Initial head (m)	*η*
1	1200	240	102.57	25.99	83.95	110	0.85
2	1320	300	0.88	0.076	0.46	115	0.86
3	560	110	2.78	1.842	2.21	85	0.87
4	600	120	164	50.6	111.1	80	0.88
5	800	160	33.5	10.4	22.00	100	0.84

**Table 3:** The optimal output range of all power plants.

Time	Plant									
	P_H,1_	P_H,2_	P_H,3_	P_H,4_	P_H,5_	P_T,1_	P_T,2_	P_T,3_	P_T,4_	P_T,5_
	(MW)	(MW)	(MW)	(MW)	(MW)	(MW)	(MW)	(MW)	(MW)	(MW)
1	[448, 465]	[508, 525]	[318, 335]	[328, 345]	[368, 385]	[708, 725]	[708, 725]	[488, 505]	[660, 660]	[400, 400]
2	[452, 469]	[512, 529]	[322, 339]	[332, 349]	[372, 389]	[712, 729]	[712, 729]	[492, 509]	[660, 660]	[400, 400]
3	[461, 484]	[521, 544]	[331, 354]	[341, 364]	[381, 404]	[721, 744]	[721, 744]	[501, 524]	[660, 660]	[400, 400]
4	[487, 508]	[547, 568]	[357, 378]	[367, 388]	[407, 428]	[747, 768]	[747, 768]	[505, 520]	[660, 660]	[400, 400]
5	[450, 465]	[510, 525]	[320, 335]	[330, 345]	[370, 385]	[710, 725]	[710, 725]	[492, 533]	[657, 660]	[397, 400]
6	[473, 490]	[533, 550]	[343, 360]	[353, 370]	[393, 410]	[733, 750]	[733, 750]	[513, 530]	[660, 660]	[400, 400]
7	[491, 510]	[551, 570]	[361, 380]	[371, 390]	[411, 430]	[751, 770]	[751, 770]	[531, 550]	[660, 660]	[400, 400]
8	[478, 498]	[538, 558]	[348, 368]	[358, 378]	[398, 418]	[738, 758]	[738, 758]	[518, 538]	[660, 660]	[400, 400]
9	[501, 520]	[561, 580]	[371, 390]	[381, 400]	[421, 440]	[761, 780]	[761, 780]	[541, 560]	[660, 660]	[400, 400]
10	[497, 517]	[557, 577]	[367, 387]	[377, 397]	[417, 437]	[757, 777]	[757, 777]	[537, 557]	[660, 660]	[400, 400]
11	[512, 531]	[572, 591]	[382, 401]	[392, 411]	[432, 451]	[772, 791]	[772, 791]	[552, 571]	[660, 660]	[400, 400]
12	[508, 529]	[568, 589]	[378, 399]	[388, 409]	[428, 449]	[768, 789]	[768, 789]	[524, 540]	[660, 660]	[400, 400]
13	[465, 482]	[525, 542]	[335, 352]	[345, 362]	[385, 402]	[725, 742]	[725, 742]	[509, 550]	[657, 660]	[397, 400]
14	[478, 497]	[538, 557]	[348, 367]	[358, 377]	[398, 417]	[738, 757]	[738, 757]	[518, 537]	[660, 660]	[400, 400]
15	[488, 507]	[548, 567]	[358, 377]	[368, 387]	[408, 427]	[748, 767]	[748, 767]	[528, 547]	[660, 660]	[400, 400]
16	[494, 515]	[554, 575]	[364, 385]	[374, 395]	[414, 435]	[754, 775]	[754, 775]	[534, 555]	[660, 660]	[400, 400]
17	[523, 545]	[583, 605]	[393, 415]	[403, 425]	[443, 465]	[783, 805]	[783, 805]	[563, 585]	[660, 660]	[400, 400]
18	[525, 545]	[585, 605]	[395, 415]	[405, 425]	[445, 465]	[785, 805]	[785, 805]	[565, 585]	[660, 660]	[400, 400]
19	[524, 545]	[584, 605]	[394, 415]	[404, 425]	[444, 465]	[784, 805]	[784, 805]	[564, 585]	[660, 660]	[400, 400]
20	[501, 510]	[561, 570]	[371, 380]	[381, 390]	[421, 430]	[761, 770]	[761, 770]	[541, 550]	[660, 660]	[400, 400]
21	[493, 512]	[553, 572]	[363, 382]	[373, 392]	[413, 432]	[753, 772]	[753, 772]	[533, 552]	[660, 660]	[400, 400]
22	[489, 509]	[549, 569]	[359, 379]	[369, 389]	[409, 429]	[749, 769]	[749, 769]	[529, 549]	[660, 660]	[400, 400]
23	[468, 488]	[528, 548]	[338, 358]	[348, 368]	[388, 408]	[728, 748]	[728, 748]	[508, 528]	[660, 660]	[400, 400]
24	[454, 473]	[514, 533]	[324, 343]	[334, 353]	[374, 393]	[714, 733]	[714, 733]	[494, 513]	[660, 660]	[400, 400]
